# Effects of Carbon Dioxide and Temperature on the Oxygen-Hemoglobin Dissociation Curve of Human Blood: Implications for Avalanche Victims

**DOI:** 10.3389/fmed.2021.808025

**Published:** 2022-02-07

**Authors:** Simon Woyke, Hermann Brugger, Mathias Ströhle, Thomas Haller, Hannes Gatterer, Tomas Dal Cappello, Giacomo Strapazzon

**Affiliations:** ^1^Department of Anaesthesiology and Critical Care Medicine, Medical University of Innsbruck, Innsbruck, Austria; ^2^Institute of Mountain Emergency Medicine, Eurac Research, Bolzano, Italy; ^3^Institute of Physiology, Medical University of Innsbruck, Innsbruck, Austria

**Keywords:** oxygen affinity, oxygen dissociation curve, carbon dioxide, temperature, avalanche burial, hypoxia, hypothermia, hypercapnia

## Abstract

Completely avalanche-buried patients are frequently exposed to a combination of hypoxia and hypercapnia with a risk of normothermic cardiac arrest. Patients with a long burial time and an air pocket are exposed to a combination of hypoxia, hypercapnia, and hypothermia which may lead to the development of the “triple H syndrome”. This specific combination has several pathophysiological implications, particularly on the cardiovascular system and oxygen transport (oxygen supply and oxygen consumption). To examine the effects on hemoglobin oxygen affinity, we investigated venous blood samples from 15 female and 15 male healthy subjects. In a factorial design of four different carbon dioxide partial pressure (PCO_2_) levels (20, 40, 60, and 80 mmHg) and five different temperature levels (13.7°C, 23°C, 30°C, 37°C, and 42°C), 30 unbuffered whole blood samples were analyzed in a newly developed *in vitro* method for high-throughput oxygen dissociation curve (ODC) measurements. P50s, Hill coefficients, CO_2_-Bohr coefficients, and temperature coefficients were analyzed using a linear mixed model (LMM). Mean P50 at baseline (37°C, 40 mmHg PCO_2_) was 27.1 ± 2.6 mmHg. Both CO_2_-Bohr (*p* < 0.001) and temperature coefficients (*p* < 0.001) had a significant effect on P50. The absolute CO_2_ effect was still pronounced at normothermic and febrile temperatures, whereas at low temperatures, the relative CO_2_ effect (expressed by CO_2_-Bohr coefficient; *p* < 0.001, interaction) was increased. The larger impact of PCO_2_ on oxygen affinity at low temperature may be caused by the competition of 2,3-BPG with PCO_2_ and the exothermic binding characteristic of 2,3-BPG. In a model of an avalanche burial, based on published data of CO_2_ levels and cooling rates, we calculated the resulting P50 for this specific condition based on the here-reported PCO_2_ and temperature effect on ODC. Depending on the degree of hypercapnia and hypothermia, a potentially beneficial increase in hemoglobin oxygen affinity in the hypoxic condition might ensue.

## Introduction

A completely avalanche-buried patient who is still able to breathe into an air pocket (any space in front of mouth and nose) may cool down during a long burial time and suffer from cardiovascular and respiratory changes that are not exclusively explainable by the effect of decreased core temperature. In the early phase of burial, acute hypoxia is associated with high carbon dioxide (CO_2_) levels due to rebreathing exhaled air (dead space ventilation) (1, 2). Without a sufficient supply of O_2_ and removal of CO_2_, hypoxia and hypercapnia increase the risk of normothermic cardiac arrest. After a long burial time but with sufficient supply or removal of respiratory gases, the patient is exposed to a combination of hypoxia, hypercapnia, and hypothermia (3). This combination of hypoxia, hypercapnia, and hypothermia has been defined as the triple H syndrome (1). Experimental studies showed that when human participants breathed into snow air pockets, the inspiratory CO_2_-fraction rapidly increased and stabilized at a level of approximately 5–6% of CO_2_ (1, 4, 5). Despite hypercapnia can speed up the cooling rate (6), the combination of hypothermia with hypoxia and hypercapnia may result in a worse neurological outcome in completely avalanche-buried patients compared to patients suffering solely from hypothermic cardiac arrest (7–9). Such outcome is determined by the temporal sequence of the events, as a decrease in oxygen supply and CO_2_ removal usually proceed the development of hypothermia.

The oxygen dissociation curve (ODC) describes the reversible binding of four molecules of oxygen to Hb (10–12). The ODC is usually described by the P50 value, that is, the value of O_2_ partial pressure, PO_2_ at which 50% of Hb is saturated with oxygen, and by the Hill coefficient (HC), a parameter that describes maximum steepness in the Hill plot and that reflects the cooperativity of ligand binding. Temperature, pH, 2,3-bisphosphoglycerate (2,3-BPG), and PCO_2_ are the four main factors that affect the ODC. Based on Severinghaus' equation, ODC should be corrected for the effects of the different factors (11). Specifically, exposing blood to CO_2_ results in a decrease of oxygen affinity *via* two ways, first a decrease in pH resulting in a decrease of oxygen affinity (Bohr effect) and second a direct binding to the oxygen-linked CO_2_ binding site and carbamino-Hb formation (CO_2_ effect) (12, 13). The combined effect with temperature as another independent factor can result in a combination of three of the four main effectors to the ODC. However, the effects of the association of hypercapnia with hypothermia on the oxygen cascade, that is, uptake, transportation, and delivery of oxygen to tissues, defined by the hemoglobin (Hb) oxygen affinity, are not fully understood. Interactions of CO_2_ and temperature may affect cellular oxygenation *via* several different pathways. In blood, the solubility of O_2_ and CO_2_ is affected by temperature and pH. In the tissues and at a cellular level, oxygen consumption and CO_2_ and also metabolic acid production decrease at lower temperatures. The possible interaction of CO_2_-Bohr coefficient (CO_2_-BC), a function of P50 CO_2_ dependency, with temperature coefficient (TC), a function of P50 temperature dependency, was studied with contradictory results in humans and animals (14–16).

The aim of this study was to describe single and combined effects of CO_2_ and temperature on the ODC in unbuffered whole blood to better understand the pathophysiology of oxygen transport in pathophysiological situations characterized by changes in temperature and levels of respiratory gases.

## Methods

This study was approved by the Ethical Board of the Medical University of Innsbruck (vote nr. 1123/2019) and is registered with clinicaltrials.gov (NCT04041531). Written informed consent was obtained from all participants.

Inclusion criteria were the age between 18 and 40 years, no recent history of acute or chronic illness, blood loss, or sojourn at high altitude (>3,000 m a.s.l.) within the past 28 days. All participants were at fasting (6 h) before blood collection. Blood was taken from nonsmoking volunteers from an antecubital vein with a minimum of stasis period. The blood samples were split into two aliquots: one was immediately analyzed by a blood gas analyzer (ABL 800 flex, Radiometer, Denmark), and another aliquot of the sample was placed on ice for the ODC experiment and analyzed within 5 h.

### ODC Experiments

Oxygen dissociation curve experiments were performed with a modification of a recently published method (17). In this method, PO_2_ is continuously decreased from 140 mmHg to 0 mmHg, and data points of PO_2_ and SO_2_ are acquired every minute to record the ODC. Within the study setting, different aliquots of the same sample can be simultaneously exposed up to four different gas mixtures. Specifically, four different PCO_2_ levels (20, 40, 60, and 80 mmHg) were applied and combined in consecutive measurements with five different temperatures (13.7, 23, 30, 37, and 42°C) that were chosen based on previously publications related to hypothermia and avalanche burial, to maximize comparability and clinical relevance (14–16, 18–20). To quantify the relative effect of temperature (TC) and CO_2_ (CO_2_-BC) on P50, coefficients were calculated [Eqs. 1, 2; (12)].


$$\begin{array}{l} Eq.1:CO_{2}-BC=\frac{\Delta \log_{10}P50}{\Delta \log_{10}PCO_{2}}\\ Eq.2:TC=\frac{\Delta \log_{10}P50}{\Delta temperature} \end{array}$$

**Figure 1:**
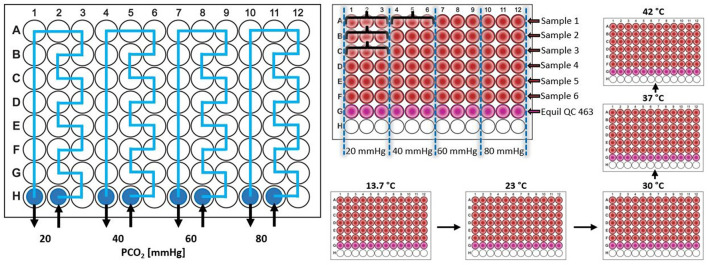
Scheme of the four channel Oxygen Dissociation Curve (ODC) plate [see (17) for further descriptions of plate design and the measurement principles]. The gas supply was randomized throughout the study, here exemplary shown for increasing PCO_2_ levels from left to right. Participant's blood samples were pipetted in rows (e.g., participant 1 in row A, participant 2 in row B, …) and triplet measurement were obtained for all combinations (e.g., for participant 1: 3 ODC measurements in wells A1, A2, and A3 for the combination of gas system 20 mmHg and applied temperature X, 3 ODC measurements in wells A4, A5, and A6 for the combination of gas system 40 mmHg and applied temperature X, …, indicated by the curly brackets). Different temperature levels were achieved by heating between the experiments, thus temperature effect measured in consecutive experiments. PCO_2_, carbon dioxide partial pressure.

In a 4 × 5 factorial design, every blood sample was exposed to all 20 combinations of four PCO_2_ levels and five temperature levels, starting with the lowest temperature level, followed by heating the entire experimental setup to the next higher temperature level. To address the increase of water vapor saturation pressure with temperature, gas mixtures were prepared volumetrically from dry, pure gases (CO_2_, N_2_ and O_2_) in gas-tight sampling bags, according to the Magnus equation (21). The temperature management of the fluorescence plate reader instrument (Tecan 200 Infinite Pro, Tecan Genios Corp., Switzerland) ensured correct temperature in the measurement chamber, whereas temperature of the conducting Viton tubes, gas mixtures, humidifiers, and temperature of the whole experimental setup was monitored and strictly controlled. The supply of the four different gas mixtures to the four gas systems in the four-channel ODC plate was randomized (Figure 1). Up to six participant's blood samples were analyzed per experiment (rows). The average of triplet measurements for every combination and sample (Figure 1) was used for analysis. Additionally, an internal standard Hb solution (Equil QC 463 Level 2, RNA medical, USA) was analyzed in each test to check and adjust for accuracy of the measurements as described before (17).

### Statistical Analysis

Student's *t*-test was used to detect sex differences in P50 and HC at baseline. Factors that affect P50, HC, CO_2_-BC, and TC were analyzed by means of a linear mixed model (LMM). The factors investigated were the following.

- For P50 and HC: sex, age (two levels: <30 and ≥30 years), temperature (13.7, 23, 30, 37, and 42°C), PCO_2_ (20, 40, 60, and 80 mmHg), and interactions of temperature with PCO_2_, sex with temperature, sex with PCO_2_, and sex with age.
- For CO_2_-BC: sex, age, temperature, and interactions of sex with temperature and sex with age.
- For TC: sex, age, PCO_2_, and interactions of sex with PCO_2_ and sex with age.

The covariance structure for the residuals of the LMM was chosen by means of the Schwarz's Bayesian Criterion among diagonal, compound symmetry, unstructured and first-order autoregression. The Holm–Bonferroni method was used to correct *p*-values for multiple comparisons. Considering ΔT as difference of temperature from 37°C (baseline temperature), ΔPCO_2_ as difference of PCO_2_ from 40 mmHg (baseline PCO_2_), and ΔP50 as difference from P50 at 37°C and 40 mmHg, a LMM was also performed to calculate an equation to predict ΔP50 from ΔT and ΔPCO_2_. For the calculation, ΔT and ΔPCO_2_ were inserted in the LMM as covariates in a model without intercept.

Data are shown as mean ± standard deviation, whereas LMM estimated means as mean (95% confidence interval; CI). *p* < 0.05 (two-sided) was considered statistically significant. Excel (Microsoft Office 2016, Microsoft Corp.) and SPSS version 25 (IBM Corp., Armonk, NY, USA) were used for data collection, calculation, and analysis.

## Results

Thirty participants (15 women and 15 men) were recruited and 30 blood samples were analyzed. Participants' mean age was 30.6 ± 3.9 years. Results of blood gas analysis, measured immediately after blood sampling, are shown in Table 1. At baseline (37°C and 40 mmHg PCO_2_), P50s in women were not significantly higher than in men (28.0 ± 2.8 and 26.2 ± 2.2 mmHg; *p* = 0.061) and HCs were 2.53 ± 0.14 and 2.56 ± 0.16 (*p* = 0.641), respectively.

**Table 1:** Results of blood gas analysis for women and men as mean ± standard deviation (Student's *t*-test).

	**All**	**Women**	**Men**	***p*-value**
pH	7.36 ± 0.03	7.37 ± 0.04	7.35 ± 0.02	0.070
PCO_2_ [mmHg]	46.5 ± 6.5	42.7 ± 5.8	50.6 ± 4.8	<0.001
HCO_3_ [mmol/l]	23.6 ± 1.5	22.9 ± 1.0	24.3 ± 1.6	0.006
Hb [g/dl]	14.9 ± 1.5	13.7 ± 0.8	16.0 ± 1.0	<0.001
Hct [%]	45.6 ± 4.5	42.0 ± 2.4	49.0 ± 3.1	<0.001
Cl^−^ [mmol/l]	106.8 ± 1.9	107.7 ± 1.5	106.1 ± 1.9	0.017
Glucose [mg/dl]	94.4 ± 6.8	91.9 ± 7.5	95.9 ± 4.6	0.094
Lactate [mg/dl]	9.9 ± 5.4	8.9 ± 5.1	10.9 ± 5.8	0.326
COHb [%]	1.4 ± 0.6	1.4 ± 0.5	1.4 ± 0.6	0.926
MetHb [%]	1.0 ± 0.1	1.0 ± 0.1	1.0 ± 0.1	0.112

*PCO_2_, carbon dioxide partial pressure; HCO_3_, bicarbonate; Hb, hemoglobin concentration; Hct, hematocrit; Cl^−^, chloride concentration; COHb, carboxyhemoglobin; MetHb, methemoglobin*.

### Factors Affecting P50

**Figure 2:**
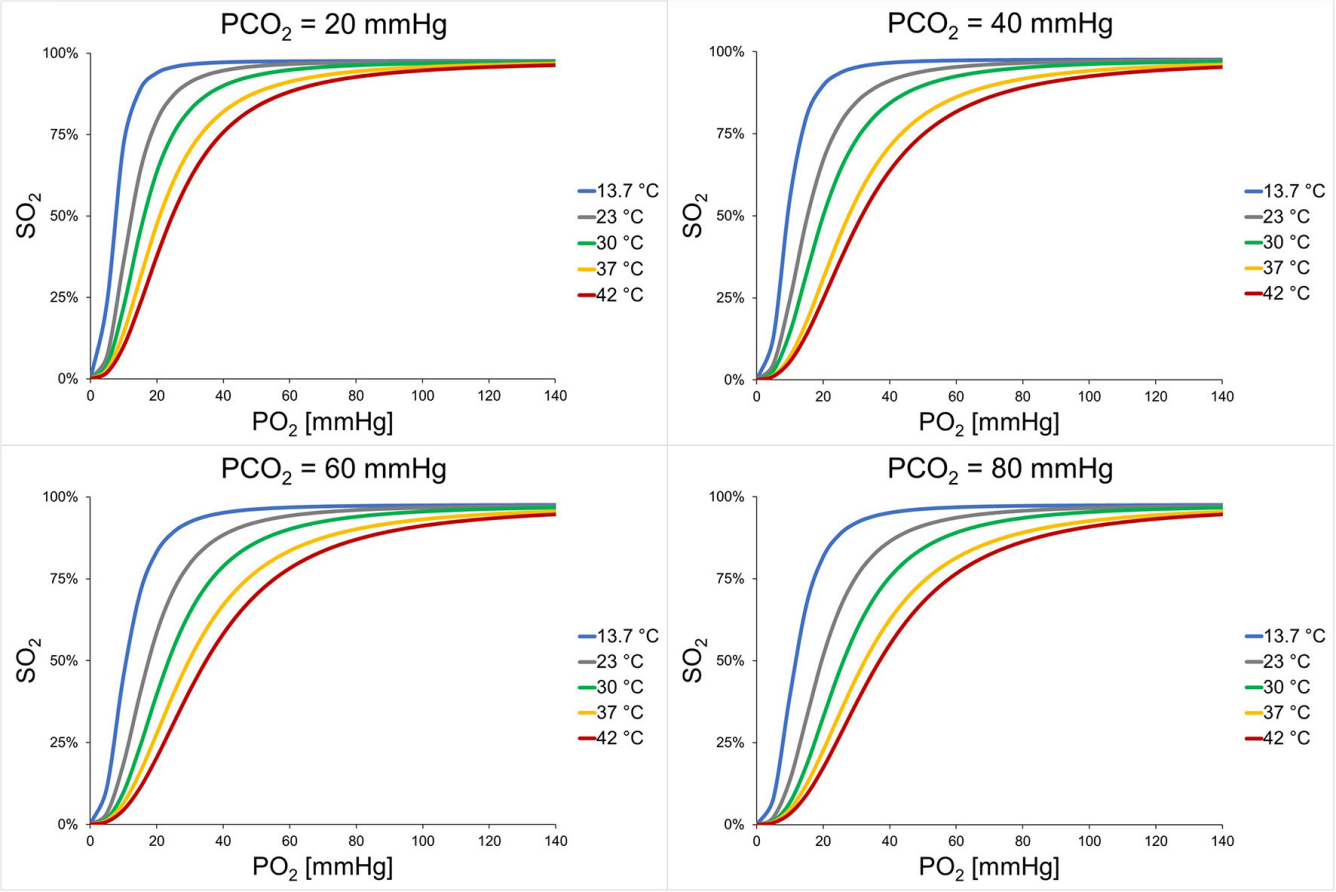
Mean ODCs of all participants. The temperature effect is shown for each PCO_2_ level. For each combination, mean P50s and mean Hill coefficient were inserted in the Hill equation [see Eq. 4 in (17)]. Each graph represents the mean of 30 participants and triplet measurements. P50, oxygen partial pressure at which 50% of hemoglobin is saturated with oxygen; SO_2_, oxygen saturation; PCO_2_, carbon dioxide partial pressure.

**Figure 3:**
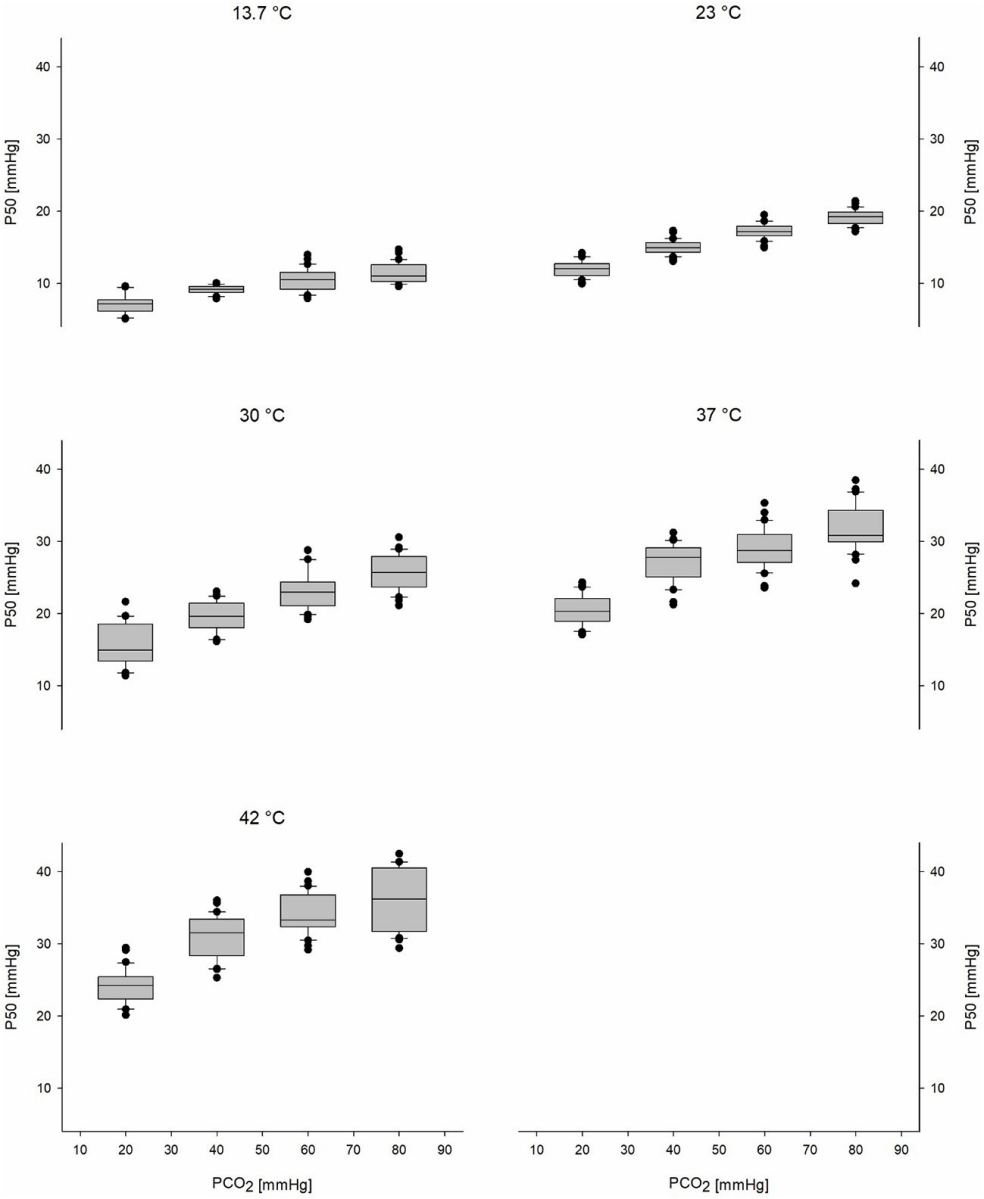
Boxplots of the P50s of all participants show the CO_2_-Bohr effect for each temperature level. Black circles represent outliers. P50, oxygen partial pressure at which 50% of hemoglobin is saturated with oxygen; PCO_2_, carbon dioxide partial pressure.

**Figure 4:**
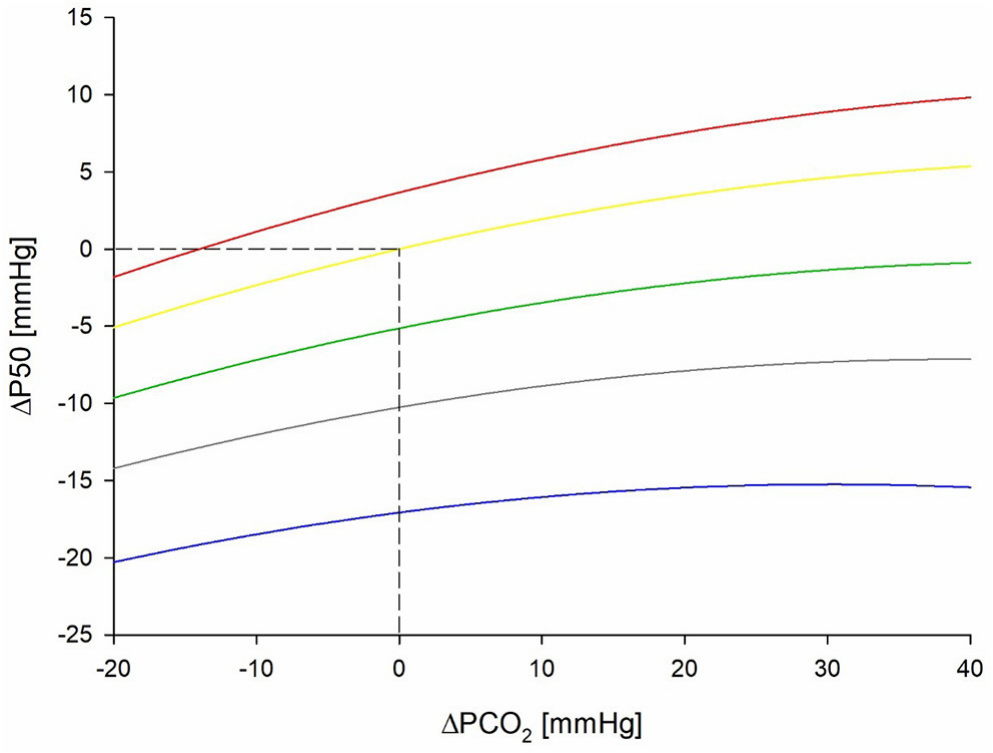
Graphical presentation in form of a nomogram for the equation of ΔP50 dependency to ΔPCO_2_ and different levels of temperature. ΔPCO_2_ denotes difference from 40 mmHg (baseline PCO_2_) and ΔP50 denotes difference from P50 at 37°C (baseline temperature) and 40 mmHg. Blue line represents 13.7°C, gray line 23°C, green line 30°C, yellow line 37°C, and red line 42°C. P50, oxygen partial pressure at which 50% of hemoglobin is saturated with oxygen; PCO_2_, carbon dioxide partial pressure.

Mean ODCs in relation to different PCO_2_ and temperature levels are shown in Figure 2. A significant effect of PCO_2_, temperature, and sex on P50 could be detected (*p* < 0.001 for all parameters; Table 2). The effect of the interaction of temperature and PCO_2_ on P50 was also significant (*p* < 0.001). At temperatures lower than 37°C, PCO_2_ increased P50 in an almost linear relationship, whereas at higher temperatures, this linearity was lost (Figure 3). An effect of age and of the interaction of sex and temperature on P50 was also significant (*p* < 0.001 and *p* = 0.001, respectively). The estimated means of P50 were higher for participants <30 years in comparison with participants ≥30 years old [21.3 (95% CI 21.1–21.5) vs. 20.7 (95% CI 20.5–20.9)]. The effect of the interaction of temperature and PCO_2_ on P50 was quantified in Eq. 3 and plotted in Figure 4.


$$\begin{array}{l} Eq.3:\Delta P50=0.733\times \Delta T+0.214\times \Delta PCO_{2}-0.002\\ \times \Delta {PCO_{2}}^{2}+0.004\times \Delta T\times \Delta PCO_{2} \end{array}$$

Δ*P50* = $P50-P50_{\left(PCO_{2}=40mmHg;temperature=37^{•}C\right)}$; Δ*PCO*_2_ = *PCO*_2_*-40 mmHg* and Δ*T* = *temperature-37*°*C*.

**Table 2:** *p*-Values of the factors inserted in the LMMs.

**Dependent variable**	**Intercept**	**Sex**	**Age**	**Temperature**	**PCO_**2**_**	Temperature ^*^PCO_**2**_	Sex ^*^Temperature	Sex ^*^PCO_**2**_	Sex ^*^Age
P50	<0.001	<0.001	<0.001	<0.001	<0.001	<0.001	0.001	1.000	1.000
Temperature coefficient	<0.001	0.576	1.000	–	0.034	–	–	1.000	0.543
CO_2_-Bohr coefficient	<0.001	1.000	0.063	<0.001	–	–	0.554	–	1.000
Hill coefficient	<0.001	0.002	1.000	<0.001	<0.001	0.002	1.000	0.096	1.000

*The p-values are corrected by means of the Holm–Bonferroni method. P50, oxygen partial pressure at which 50% of hemoglobin is saturated with oxygen; PCO_2_, carbon dioxide partial pressure*.

* *An asterisk between two factors indicates the effect of interaction of the two factors. - A dash indicates that the factor was not inserted in the LMM*.

### Factors Affecting CO_2_-Bohr Coefficient

**Figure 5:**
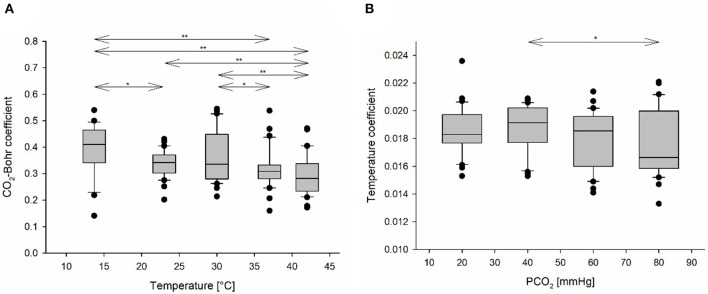
**(A)** CO_2_-Bohr coefficients (CO_2_-BCs) in relation to temperature, presented as boxplots. CO_2_-BC, a measure of relative CO_2_-Bohr effect, decreases with increasing temperature. **(B)** Temperature coefficients (TCs) in relation to PCO_2_, presented as boxplots. Black circles represent outliers. A line with arrows and asterisks indicates a pairwise comparison which was detected as statistically significant by the LMM; one asterisk denotes 0.05 ≤ p < 0.1, and two asterisks denote *p* < 0.05.

With LMM, an effect of temperature on CO_2_-BC could be detected (*p* < 0.001; Table 2). CO_2_-BCs progressively decreased with the increase of temperature (Figure 5A). The differences were significant between 13.7 and 37°C (*p* = 0.001), 13.7 and 42°C (*p* < 0.001), and also between 23 and 42°C (*p* = 0.044) and between 30 and 42°C (*p* = 0.001).

### Factors Affecting Temperature Coefficient

Only an effect of PCO_2_ (*p* = 0.034; Table 2) was detected on TC. There was an indication that TCs at 40 mmHg (baseline PCO_2_ level) were higher than at 80 mmHg PCO_2_ (*p* = 0.051; Figure 5B).

### Factors Affecting Hill Coefficient

**Figure 6:**
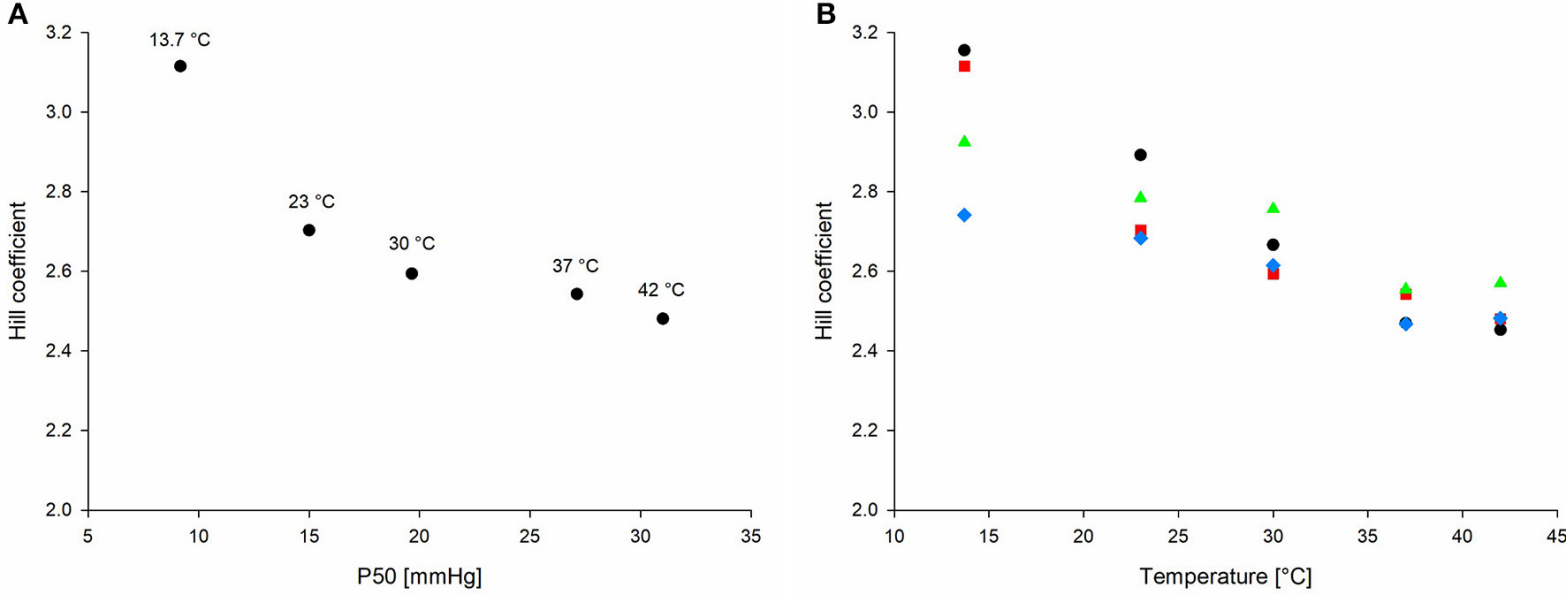
**(A)** Correlation of Hill coefficient (HC) with P50. HCs decrease with increasing P50. **(B)** Mean Hill coefficient over all 20 combinations of PCO_2_ and temperature (black circle 20 mmHg, red square 40 mmHg, blue rhombus 60 mmHg, and green triangle 80 mmHg). P50, oxygen partial pressure at which 50% of hemoglobin is saturated with oxygen.

There was an effect on HC by PCO_2_, temperature, sex, and the interaction of temperature and PCO_2_ (*p* ≤ 0.002 for all parameters; Table 2). HCs were different at different P50s (Figure 6A) with a typical flattening of the ODC when shifted to the right. Mean HCs changed in relation to the different combinations of PCO_2_ and temperature (Figure 6B). Overall, HCs were higher at PCO_2_ 80 mmHg compared to PCO_2_ 60 mmHg for a specific temperature (i.e., a right-shifted, but steeper ODC). The estimated mean of HC at 60 mmHg was significantly lower in comparison with 20, 40, and 80 mmHg [2.73 (95% CI 2.66–2.80) at 20 mmHg; 2.69 (95% CI 2.65–2.72) at 40 mmHg; 2.60 (95% CI 2.56–2.63) at 60 mmHg; and 2.72 (95% CI 2.68–2.75) at 80 mmHg] (i.e., an extended ODC shape).

## Discussion

We describe a significant interaction of the CO_2_-Bohr effect and the temperature effect on the ODC in unbuffered whole blood samples. At temperatures lower than 37°C, the effect of temperature seems to outweigh the effect of even high CO_2_ levels. There was a left-shifted ODC that could facilitate oxygen uptake in the lungs but might possibly limit deoxygenation at tissue level. Such modification could be protective against excessive hypoxia in a state of hypercapnia and hypothermia, as in the triple H syndrome. At temperatures higher than 37°C and high CO_2_ levels, the effect of CO_2_-BC on the ODC decreased. Such findings may be due to an excess of respiratory gases (i.e., saturation effect). An increase in PCO_2_ up to 80 mmHg resulted in right-shifted but also slightly steeper ODCs compared to those at PCO_2_ 60 mmHg, indicated by higher HCs, for example, at 42°C. Due to the great extent of both effects, this finding might be of clinical impact for the oxygen transport from the lungs to the tissue in hypoxic situations where both parameters are out of physiological range and pulmonary oxygen supply, oxygen transport capacity, or cardiac output is deteriorated (e.g., critical illness, acute respiratory distress syndrome, COVID-19, sepsis, and hypothermic major trauma).

Our data showed that CO_2_-Bohr effect decreased with increasing temperatures from hypothermic to normothermic conditions which is supported by some of the available literature. Differences in the methodological approaches and results in studies investigating the interaction of the CO_2_-Bohr and temperature effect in human and animal studies caused inconsistencies. In 1961, Callaghan studied blood of dogs under hypothermic conditions (15, 23, 30, and 37°C) at various CO_2_ levels (20, 40, 60, and 70 mmHg) with a self-constructed instrument (16). When analyzing their published data using Eq. 1, CO_2_-Bohr effect showed a decrease with increasing temperatures from hypothermic to normothermic conditions. Such results are consistent with our data in humans. In 1977, Hlastala studied the interactions between all four main parameters affecting ODC on 93 humans (14) using an instrument presented by Duvelleroy (22). They also studied different combinations of temperature (23, 30, 37, and 44°C) and CO_2_ levels (2.5, 5.8, and 8.5%). Hlastala reported an even increased CO_2_-Bohr effect with increasing temperature from hypothermic to normothermic conditions. In 1980, Reeves also studied temperature levels from 13 to 43°C and CO_2_ levels from 1 to 8% (15) using a self-constructed instrument for rapid ODC measurements in a blood film (23). Reeves reported neither variations in the shape of the ODC with temperature nor an interaction of CO_2_-Bohr effect with temperature and also rejected the finding reported by Hlastala that the TC varies with oxygen saturation (15). Overall, we assume that the main reason for the contradictory results is the different approaches in calculating the CO_2_-BC. Hlastala calculated CO_2_-BC using pH and base excess. pH and base excess are not only difficult to be exactly measured in a blood film, but pH itself is temperature-dependent by definition (24). In our study, only PCO_2_ values were used for calculations. Unfortunately, raw data are not published by Hlastala, so a direct comparison by recalculating their results using our equation is not possible. Hlastala also used an oxygenation protocol, whereas both Callaghan (15) and our group worked with deoxygenation for the determination of ODCs (17). As the solubility of CO_2_ increases with a decrease of temperature, and CO_2_ and O_2_ are competing agents in binding to hemoglobin (Haldane effect), the way of the oxygen ramp might be of importance as this could interfere with the measurement results. Both methods are working with blood films, and thus, the impact of solubility changes due to temperature changes should be neglectable. Our method might be of advantage for clinical insights when compared to an oxygenation protocol. It simulates more the pathological changes, as at the beginning, full saturation is expected.

Our data showed that the effect of CO_2_ was diminished at higher CO_2_ levels and the relative effect, expressed by the CO_2_-BC, decreased with increasing temperature. Benesch showed that the binding of 2,3-BPG to hemoglobin is an exothermic reaction (25), reducing the amount of 2,3-BPG bound to hemoglobin at higher temperatures. CO_2_ binding to hemoglobin and carbamino group formation is favored, as 2,3-BPG and CO_2_ are competing agents to the binding to Hb (13, 14). Such data suggest that there is a strong competition between CO_2_ and 2,3-BPG at lower temperatures, resulting in a strong effect of PCO_2_ on P50. Conversely, at higher temperatures (e.g., 42°C), the majority of 2,3-BPG is dissociated from hemoglobin, lowering the impact of the competition with CO_2_, and emphasizing the effect of lower PCO_2_ levels (e.g., 20 mmHg or 40 mmHg). A further increase of PCO_2_ (60 mmHg or 80 mmHg) seems to cause a reduction of the impact on P50, because the N-terminal binding sites of the Hb β-chains may be already occupied by CO_2_ (26). Christiansen described that at lower PCO_2_ levels, CO_2_ uptake is facilitated, whereas at higher PCO_2_ levels, the curve flattens and may indicate a further increase of CO_2_ uptake only by physically solved CO_2_ (26). As Hb binding sites for CO_2_ are limited by the amount of Hb, we assume that at some point, a state of saturation in the combination of CO_2_ and O_2_ binding to Hb exists, and a further increase in PCO_2_ does not lead to an additional right-shift of the ODC. The human body might not benefit of an improved oxygen delivery to the tissue at P50 levels exceeding 40 mmHg. If the oxygen affinity of Hb is too low, a sufficient binding and thereby transportation of oxygen cannot be provided any more.

Böning also reported an interaction of the Bohr effect and TC and a sex dependency of both effects regarding P50 (27). Our data also show that the temperature effect on P50 is sex-dependent, but the difference almost disappeared at temperature <23°C.

When oxygen content in the inspiratory air is limited, a left-shifted ODC, which increases oxygen uptake in the lungs, is beneficial. For instance, P50 in humans exposed to extreme altitude is reduced due to hypocapnia, resulting in increased SO_2_. In the placenta, PaO_2_ is very low (30 mmHg) and ODC of the fetus is shift to the left due to fetal hemoglobin (P50 about 20 mmHg) (28). Such status can be observed also in accidentally hypothermic patients and can explain better outcome after hypothermic cardiac arrest. Completely avalanche-buried patients with long burial can develop accidental hypothermia, but they are also exposed to a combination of hypoxia and hypercapnia that can lead to a worse outcome (2, 24). Human experimental studies showed that smaller air pockets and higher density of the surrounding avalanche debris can impair respiratory gas supply or removal and lead to a more severe level of hypoxia and hypercapnia (1, 4, 5). The effects of the combination of the three factors could only be tested in animal models (29, 30). A porcine study on the triple H syndrome experimentally showed a decrease in brain tissue PO_2_, cerebral venous SO_2_ and regional cerebral SO_2_ at a core temperature of 28°C. The reduced FiO_2_ and increased CO_2_ caused a mismatch between metabolic oxygen consumption and oxygen delivery (30). In sole deep hypothermia, cerebrovascular reactivity (cerebral autoregulation) was impaired in pigs (31). Interestingly, whereas mean arterial pressure and cerebral perfusion pressure were decreased at 28.8°C, brain oxygenation was increased (31). Our study suggests that there can be an ODC shift to the left in spite of moderate elevated CO_2_ level, due to hypothermia.

Based on measured CO_2_ levels from a human experimental study (4) and cooling rates from observational studies of avalanche victims (32–34), the expected P50s in avalanche-buried victims were simulated in a model (Table 3). The calculated P50 (Table 3, P50 at 36.4°C and PCO_2_ of 47 mmHg) suggests an increased tissue oxygen unloading that might contribute to outcome (e.g., better cerebral oxygenation). Such status could be found in the early stage of triple H syndrome (5) when the patients is still not hypothermic. In the presence of an air pocket and sufficient exchange of respiratory gases, arterial PCO_2_ might remain stable, whereas the progressive cooling could allow a further left-shift (i.e., a core temperature <35°C; Table 3, P50 at 34°C or 28°C and PCO_2_ of 47 mmHg). In case of insufficient exchange of respiratory gases, there could be higher PaCO_2_ levels (e.g., 75 mmHg, see Table 3) without a left-shift.

**Table 3:** A model for P50 in an avalanche burial model.

	**PCO**_**2**_		
	**41 mmHg**	**47 mmHg**	**75 mmHg**
28°C	20.7	21.7	24.3
34°C	25.1	26.2	29.5
36.4°C	26.9	28	31.6

*Calculations based on mean P50 of this study (P50 = 27.1 mmHg) with PCO_2_ levels (4) and cooling rates (32–34) from other studies, respectively. Temperature is denoted in columns and PCO_2_ in rows. P50, oxygen partial pressure at which 50% of hemoglobin is saturated with oxygen; PCO_2_, carbon dioxide partial pressure*.

In other hypothermic patients, oxygen consumption is decreased and thereby also CO_2_ production. The relevance of the interaction of PCO_2_ and temperature may thus also have an impact of the fields of therapeutic hypothermia, ARDS, pulmonary oedema, in cardio-anesthesiology, and intensive care medicine. In hypothermic circulatory arrest, acid-base management is based on two different approaches: pH-stat or alpha-stat. Whether one of the two approaches is superior to the other is under debate, yet an age-dependent approach seems preferable (35). The present findings of an interaction effect of PCO_2_ and temperature on the ODC might add a small piece of the puzzle in this debate. In an in-hospital setting, P50 as a therapeutic target plays nowadays a tangential role, despite possible treatment options (36, 37).

### Limitations

CO_2_-BC was established as a function of PCO_2_, as pH is temperature-dependent and hard to measure in a small volume of blood sample, whereas PCO_2_ is a parameter measured routinely *in vivo* in a clinical setting. Due to the small volumes of blood (15 μl) in single cell layer films, there was neither a pH measurement nor a measurement of base excess feasible. Thus, a definition and calculation of CO_2_-BC based on pH and base excess could not be carried out, which results in a limitation of comparability to other published data (14). The supply of the different PCO_2_ levels to the 3H plate was randomized, but we started always with the lowest temperature and then increased the temperature for practical reasons. Thus, at lower temperature, the blood was fresher than at higher temperature. This possible bias should be neglectable as the correct storage of the blood samples on ice was a main focus and the metabolic parameters were shown to remain stable within hours after blood sampling (17). The presented equation that can be used for exact P50, PCO_2_, and temperature correction is obtained from one sample of participants (*n* = 30), and another independent study evaluating this interaction is necessary to validate the proposed equation. In this study, the interactions of CO_2_-BC and TC were studied in an *in vitro* experiment. Direct transfer of study findings in an *in vivo* scenario should be done cautiously. Due to the difficulties in feasibility and transfer from animal studies (38–41), the *in vitro* ODC determination is advantageous under these conditions. Further studies are needed to investigate the reasons for the here-reported interaction of CO_2_-Bohr effect with temperature effect. The saturation dependency of Bohr effect (42) was not accounted for in this study.

## Conclusion

We describe a significant interaction of the CO_2_-Bohr effect and the temperature effect on the ODC in unbuffered whole blood samples. At temperatures lower than 37°C, the effect of temperature seems to outweigh the effect of even high CO_2_ high levels. There was a left-shifted ODC that could result in an increased oxygen uptake in the lungs. At temperatures higher than 37°C and high CO_2_ levels, the effect of CO_2_-BC on the ODC decreased. Depending on the degree of hypercapnia and hypothermia in a completely avalanche-buried patient, there could be a left-shift of the ODC with a potential benefit when oxygen content in the inspiratory air is limited (i.e., hypoxia and hypercapnia) and oxygen consumption of the brain is decreased (i.e., hypothermia). Further studies are needed to investigate *in vivo* the current *ex vivo* results and the systemic effects of the reported interaction effect of CO_2_-Bohr effect and TC, and the clinical use of P50 could allow to better triage avalanche.

## Data Availability Statement

The raw data supporting the conclusions of this article will be made available by the authors upon reasonable request.

## Ethics Statement

The studies involving human participants were reviewed and approved by Ethikkommission der Medizinischen Universität Innsbruck, Innrain 43, Innsbruck, Austria. The participants provided their written informed consent to participate in this study.

## Author Contributions

SW prepared original draft. SW and TDC conducted data curation, formal analysis, and visualization. HB, MS, TH, and GS have done supervision and project administration. All authors performed conceptualization, methodology, validation, involved in writing, reviewing, editing, and approved the submitted version.

## Funding

This study was funded by equity capital of the Medical University of Innsbruck, Austria, and Eurac Research, Bolzano, Italy.

## Conflict of Interest

The authors declare that the research was conducted in the absence of any commercial or financial relationships that could be construed as a potential conflict of interest.

## Publisher's Note

All claims expressed in this article are solely those of the authors and do not necessarily represent those of their affiliated organizations, or those of the publisher, the editors and the reviewers. Any product that may be evaluated in this article, or claim that may be made by its manufacturer, is not guaranteed or endorsed by the publisher.
